# Measurement of Agricultural Green Development Level in the Three Provinces of Northeast China Under the Background of Rural Vitalization Strategy

**DOI:** 10.3389/fpubh.2022.824202

**Published:** 2022-03-11

**Authors:** Dainan Hou, Xin Wang

**Affiliations:** ^1^School of Business, Minnan Normal University, Zhangzhou, China; ^2^College of Life Science, Longyan University, Longyan, China; ^3^Fujian Provincial Key Laboratory for the Prevention and Control of Animal Infectious Diseases and Biotechnology, Longyan, China; ^4^Key Laboratory of Preventive Veterinary Medicine and Biotechnology, Longyan University, Longyan, China; ^5^Chinese International College, Dhurakij Pundit University, Bangkok, Thailand; ^6^Fujian Jonorm Bio-Pharmaceutical Co., Ltd., Longyan, China

**Keywords:** rural vitalization strategy, agricultural green development level, entropy-gray correlation, three northeastern provinces, China

## Abstract

This paper offers an evaluation index system of agricultural green development level based on the Rural Vitalization Strategy. The evaluation index system includes four dimensions: agricultural development, ecological resource protection, environment-friendly, and industrial extension and integration. Then, the paper calculates the level of agricultural green development in the three provinces of Northeast China using the entropy-gray correlation method from 2009 to 2019. The calculation results show that between 2009 and 2019, the level of agricultural green development in the three northeastern provinces of China fluctuates and increases, and there is some variation between them: in terms of the overall level of agricultural green development Heilongjiang Province > Liaoning Province > Jilin Province, in terms of the average change speed of agricultural green development level, Heilongjiang Province > Liaoning Province > Jilin Province; in terms of each dimension, the average level of correlation degree of agricultural development dimension is Liaoning Province > Heilongjiang Province > Jilin Province, the average level of correlation degree of ecological resource protection dimension is Heilongjiang Province > Jilin Province > Liaoning Province, the average level of correlation degree of environment-friendly dimension is Heilongjiang Province > Jilin Province > Liaoning Province, and the average level of correlation degree of industrial extension and integration is Liaoning Province > Heilongjiang Province > Jilin Province. Finally, the basis of the calculation results, combined with the actual situation of agricultural development in the three provinces, the countermeasures, and suggestions for promoting the green development of agriculture are put forward. Specifically, it includes the establishment of an evaluation system for agricultural green development; strengthening the protection of agricultural ecological resources; increasing rural environmental governance; promoting the integrated development of rural tertiary industries; vigorously supporting agricultural scientific and technological innovation; and strengthening regional cooperation and other suggestions.

## Introduction

This study is aimed to build an evaluation index system for the level of green development of agriculture in China under the background of rural revitalization, evaluate the level of green development of agriculture in the three northeast provinces, and put forward countermeasures and suggestions. The objective of this study is to measure the level of agricultural green development in the three northeastern provinces of China as objectively as possible through a suitable methodology and the right evaluation index system and then to propose countermeasures. The issue of evaluating the level of agricultural green development is a very important one. Agriculture is the source of food and clothing for human society, the foundation of survival, and an important pillar that guarantees the development of the national economy and social progress. In recent years, my country's agriculture has developed rapidly. At the same time, in the process of agricultural production, the unreasonable use of pesticides, chemical fertilizers, mulching film, etc., the random discharge of livestock and poultry manure, rural production and household garbage, crop straws, and other wastes have brought negative impacts on the rural environment and seriously, seriously threatening the sustainable development of the rural economic and social environment, and also threatened people's food and water sources. Therefore, promoting the green development of agriculture can not only effectively solve the “high consumption” and “high pollution” caused by agricultural production in the past and promote the profound transformation of agriculture from “quantity” to “quality” but also fully implement the concept of green development and realize the inevitable choice of rural revitalization. At the same time, this is also an objective requirement forced by the natural development of agriculture (1), and green agriculture is the foundation of human nutrition and health.

Since the 18th National Congress of the Communist Party of China, the Party Central Committee and the State Council had attached great importance to green development. In the report on the 19th National Congress of the Communist Party of China, “persist in the harmonious coexistence of man and nature” was incorporated into the socialist ideology and basic strategy with Chinese characteristics in the new era and proposed the implementation of a Rural Revitalization Strategy. In the “Opinions of the Central Committee of the Communist Party of China and the State Council on the Implementation of the Rural Revitalization Strategy” issued by the “No. 1 Central Document” on January 2, 2018, comprehensive deployment of the Rural Revitalization Strategy was carried out. On September 26 of the same year, the State Council issued the “Strategic Plan for Rural Revitalization (2018–2022).” In the plan, it is proposed that “to be guided by eco-environmental friendliness and sustainable use of resources, promote the formation of green agricultural production methods, realize input reduction, clean production, waste recycling, collocation of industrial models, and improve the ability of sustainable agricultural development. The first year of China's “14th Five-Year Plan” is 2021. It is a year to consolidate and expand the results of poverty alleviation and accelerate the promotion of rural revitalization. On February 21, 2021, the central government issued the “No. 1 Document” “Opinions of the Central Committee of the Communist Party of China and the State Council on Comprehensively Promoting Rural Revitalization and Accelerating Agricultural and Rural Modernization.” The major arrangements made by the Party Central Committee and the State Council put forward the overall requirements for promoting green agricultural development in the document.

The green development of agriculture is an important guarantee of realizing the prosperity of agricultural and rural industries, ecological livability, and affluent life. It is an important theoretical and practical topic. Therefore, in the new stage, under the background of comprehensively promoting rural revitalization and accelerating agricultural and rural modernization, it is of great practical significance to study the issue of agricultural green development.

The contributions of this paper to the existing literature are as follows. First, different from the traditional perspective, from the perspective of Rural Revitalization Strategy, we attempt to construct an indicator system for the evaluation of the level of agricultural green development from the four dimensions of agricultural development, ecological resource protection, environmental friendliness, and industrial extension and integration. The index system can be applied to evaluate the agricultural green development level of provinces, cities, and counties in China before and after the implementation of the Rural Revitalization Strategy and provide a decision-making basis for governments at all levels. At the same time, the index system can provide a reference for scholars to carry out similar research. Second, different from previous studies, we focused on the research of regional agricultural green production issues. Therefore, the three provinces of Northeast China, which are geographical units with similar climatic conditions, agricultural production conditions, similar geo-cultures, and close economic and social ties, are selected as the research area. The three northeastern provinces have similar natural and climatic conditions and agricultural production conditions and are important commodity grain bases and livestock and poultry product breeding bases in China. Third, this paper uses short-panel data, and the sample size is limited. Thus, the entropy method-gray correlation evaluation method is selected. Objective weighting through the entropy method can remove subjective influence (2). The gray relational analysis method can deal with the incompletely clear gray system, and the evaluation accuracy of the small sample index is relatively high (3). Therefore, the application of this method for the evaluation in this paper can improve the accuracy of the evaluation.

The remainder of this study is organized as follows. Section literature review reviews the literature review. Section materials and methods introduces the data and research methods. Section results and discussion discusses the empirical results. Section conclusions and policy implications shows conclusions and makes policy recommendations.

## Literature Review

The development of green agriculture is an important direction for the future development of agriculture in China and the world. To better realize the green development of agriculture, it is necessary to study the theory and practice of agricultural green development. Scholars have achieved certain results in the research of agricultural green development. Specifically, they mainly include the following three aspects:

First, in terms of theoretical research on agricultural green development, there are mainly the following viewpoints. In the term of agricultural green development, “agriculture” is the main body, “development” is the core, and “green” is the method and goal. Achieving green agricultural development is not only an important breakthrough to solve the pressure on the ecological environment and resource shortages of China's agricultural development, but also an objective requirement to meet the people's growing needs for a better life (4). The agriculture green development is a necessary condition and fundamental guarantee for social and economic stability and progress and sustainable human survival and reproduction (5), and it is the specific application of the Chinese Communist Party's coordinated and sustainable development concept in the agricultural field (6). The core and key of agricultural green development is the protection of water and soil resources, which is the basis for ensuring the quality and safety of agricultural products (7). Although agricultural green development includes multiple goals and stakeholders and involves multiple links in the food system, in essence, “green” and “development” are the keys to agricultural green development, and the relationship between the two should conform to the Kuznets curve (8).

Second, the research literature is mainly the research on the evaluation of agricultural green development. To evaluate the agricultural green development in different provinces and cities of China, Zhou et al. (9) constructed an indicator system from the three dimensions of high-efficiency agriculture, green production, and integrated development and evaluated the green development of agriculture in Shaanxi Province from the perspective of rural revitalization. Fu et al. (10) analyzed green agriculture development in Zhejiang Province from the perspective of agricultural resource utilization. Some researchers used the entropy method to evaluate the level of agricultural green development in Anhui province (11), major grain-producing regions of China (12), Qinghai Province (13), and Jiangsu Province (14). Wang et al. (15) based on the input-output index system of agricultural green production, Slacks-based model (SBM) was adopted to measure the agricultural green production efficiency of 31 prefecture-level cities in the middle reaches of the Yangtze River from 2008 to 2018, and the Tobit model of panel fixed effect was used to analyze the driving effect of external factors that affect the agricultural green production efficiency of urban agglomeration in the middle reaches of the Yangtze River. To evaluate the agricultural green development of China, Tu (16) used data envelopment analysis (DEA) research ideas and methods to calculate the agricultural green development index, analyzing the level of China's agricultural green development from both static and dynamic aspects, and based on variable coefficients semi-parametric estimation, analyzed the important determinants of agricultural green development and the influence of regional heterogeneity on agricultural green development. Liu (17) used the minimum spanning tree to analyze the agricultural green development index of 30 provinces in China. Gong et al. (18) constructed an indicator system that consists of 10 indicators in three dimensions, low-carbon production, economic growth, and safe supply, and used the analytic hierarchy process to measure and compare China's agricultural green development index from 2005 to 2018. Liu et al. (19) constructed an evaluation index system from five dimensions, supply capacity, resource utilization, environmental quality, ecosystem maintenance, and farmers' lives, to evaluate the level of green development of agriculture in China and study its vertical and spatial evolution.

Third, the research literature is the research on the countermeasures of agricultural green development. Agricultural green development is a profound change in the concept of development and promoting agricultural green development will be an important measure to break through the bottleneck of China's agricultural development and achieve the goal of agricultural modernization (20). Scholars proposed to establish a basic database of relevant indicators of agricultural green development (8, 20), improve agricultural production conditions, increase agricultural technology content (21), and strengthen agricultural non-point source pollution treatment and restoration (22, 23), strengthen the coordinated development of the economic belt and the economic development zones of the provinces and cities, adjust the agricultural layout (24) to establish a green consumer market with premium functions (25), and emphasize the importance of rural finance to the green development of agriculture supporting and guiding role (26), strengthening the role of the government, i.e., subsidies for agricultural green production, and implementing punishment mechanisms (27).

Judging from the previous studies, scholars have researched issues, such as agricultural green development theory, agricultural green development effect evaluation, and agricultural green development countermeasures, providing theoretical basis and development ideas for the study of agricultural green development.

However, there is still potential for further research. First of all, most of the previous theoretical studies on agricultural green development are based on agriculture, ecology, resources, and other aspects to explore the connotation of agricultural green development, lacking consideration of the strategic background of rural revitalization.

Second, the evaluation indexes system of previous studies lacks consideration of environmental and ecological problems. In terms of evaluation methods, the entropy method and analytic hierarchy process are mainly used alone. These methods have some limitations when used alone, which may affect the accuracy of evaluation results. In addition, some scholars use the DEA method to calculate based on input and output from the perspective of agricultural green production efficiency. It is not appropriate and sufficient to analyze the problem of agricultural green development only from the perspective of agricultural green production efficiency.

Thirdly, most of the previous studies are based on the scope of the whole China or a province or a city, and there are few in-depth studies on a certain region, especially in areas with similar climate characteristics, agricultural production conditions, and planting and breeding structure.

Based on this, this study selects three northeastern provinces (Heilongjiang, Jilin, and Liaoning) with similar natural environmental characteristics as the research object and constructs an evaluation index system from four dimensions, agricultural development, ecological resource protection, environmental friendliness, and industrial extension and integration, using the improved entropy weight-gray correlation method to evaluate the level of agricultural green development in the three northeastern provinces, and put forward policy recommendations on this basis.

## Materials and Methods

### Materials

#### Study Area

The three northeastern provinces of China, namely, Heilongjiang Province, Jilin Province, and Liaoning Province, are located between 38°43′ and 53°24′ N and 115°20′ E and 135°05′E. The region is bordered by the Inner Mongolia Autonomous Region in the west, Hebei Province in the south, Shandong Province across the sea, and North Korea and Russia in the east. The total area of the region is about 791.8 thousand km^2^ (28).

The three northeastern provinces of China have various types of landforms, surrounded by mountains and rivers, and fertile fields. Plateaus, plains, and mountains are widely distributed, suitable for diversified agricultural operations. The soil is fertile, and black soil and chernozem are widely distributed, making it one of the three major fertile black soil distribution areas in the world. The three northeastern provinces have a temperate monsoon climate, with strong solar radiation, high summer temperatures, moderate precipitation, rain and heat in the same season, and large temperature differences between day and night, which are beneficial to agricultural production.

The characteristics of agricultural planting in the three northeast provinces are shown in Table 1. In addition, its main crops include rice, summer corn, spring wheat, and soybeans. In 2019, the grain output of the three northeastern provinces reached 11.623.9 million tons, accounting for 20.80% of China's national grain output. They are China's important commodity grain bases. At the same time, its internal geographical division of labor is obvious, the economic system is complete, the geographical culture is similar, and the economic and social connections are close. It is a relatively complete economic zone and geographical unit in China. Therefore, the selected research areas are the three provinces of Northeast China, to measure the level of agricultural green development in the three provinces of Northeast China, and put forward corresponding countermeasures and suggestions on this basis.

**Table 1:** Characteristics of agricultural planting in the three northeast provinces.

**Project**	**Specific situation**
Farming system	One ripe a year
Agricultural activities	Spring sowing, summer tube, autumn harvest
Crop varieties	Prefer cool and cold crops
Crop growth cycle	April to October

#### Data

The data used in this research comes from the “China Agricultural Yearbook,” “China Rural Statistical Yearbook,” “China Environmental Statistics Yearbook,” “Green Food Statistical Yearbook” (2009–2019), “Statistical Bulletin” (Heilongjiang, Jilin, Liaoning), 2010-2020, “Township and Village Enterprises,” “China Agricultural Products Processing Industry Yearbook,” etc. Some indicators are obtained directly, and some indicators are obtained through calculations.

#### Construction of Evaluation Index System for Agricultural Green Development Level

Based on the research on the connotation of agricultural green development, this paper conducts in-depth research “Strategic Plan for Rural Revitalization (2018–2022),” “Technical Guidelines for Agricultural Green Development (2018–2030),” “The Fourteenth Five-Year Plan for the National Economic and Social Development of the People's Republic of China and the Outline of Long-term Goals for 2035,” “No. 1 Central Document” in 2021, and other important plans and documents require for the agriculture green development. At the same time, the principles of constructing an evaluation index system that takes into account comprehensiveness, scientificity, orientation, and operability are taken into account. Therefore, as shown in Table 2, 16 indicators are selected from four dimensions, agricultural development, ecological resource protection, environment-friendly, and industrial extension and integration, to build an evaluation index system for the level of green development in agriculture. The index system contains the new requirements of the Rural Revitalization Strategy for the green development of agriculture.

**Table 2:** Evaluation index system of agricultural green development level.

**First-level indicator**	**Second-level indicator**	**Description**	**Unit**	**Indicator attribute**
Agricultural development (A_1_)	Per capita agriculture, forestry, animal husbandry and fishery output value (B_1_)	Agriculture, forestry, animal husbandry and fishery total output value /agricultural population	yuan/person	+
	Unit planting area yield of grain (B_2_)	Grain yield/grain sown area	ton/ha	+
	Unit planting area total agricultural output value (B_3_)	Total agricultural output value/grain sown area	10^4^yuan/ha	+
	Unit planting area total power of agricultural machinery (B_4_)	Total power of agricultural machinery/grain sown area	kW/ha	+
	Labor productivity (B_5_)	Total output value of agriculture, forestry, animal husbandry and fishery/employees in the primary industry	10^4^yuan/person	+
Ecological resource protection (A_2_)	Forest coverage rate (B_6_)	Directly obtained from the statistical yearbooks	%	+
	Area of soil erosion under control (B_7_)	Directly obtained from the statistical yearbooks	10^3^ha	+
	Water-saving irrigation efficiency (B_8_)	Water-saving irrigation area/grain sown area	%	+
Environment-friendly (A_3_)	Pesticide application intensity (B_9_)	Total pesticide application amount/total grain sown area	ton/ha	-
	Fertilizer application intensity (B_10_)	Total chemical fertilizer application amount/total grain sown area	ton/ha	-
	Agricultural film application intensity (B_11_)	Total agricultural film application amount/total grain sown area	ton/ha	-
	Popularization rate of rural sanitary toilets (B_12_)	Obtained directly from the statistical yearbooks	%	+
	Number of nature reserves at all levels (national and provincial) (B_13_)	Obtained directly from the statistical yearbooks	pcs	+
Industrial extension & integration (A_4_)	Number of green food labels unit planting area (B_14_)	Number of green food certified in the current year/grain sown area	number/ha	+
	Engel coefficient of rural residents (B_15_)	Obtained directly from the statistical yearbooks	%	-

The agricultural development dimension reflects the fundamentals of agricultural production and reflects economic benefits. Specifically, it includes per capital agriculture, forestry, animal husbandry and fishery output value, unit planting area yield of grain, unit planting area total agricultural output value, unit planting area total power of agricultural machinery, and labor productivity five secondary indicators. The dimension of ecological resource protection is the core of agricultural green development and sustainable agricultural development. It reflects ecological benefits, such as three secondary indicators of forest coverage, area of soil erosion under control, and water-saving irrigation efficiency. Environment-friendly is the fundamental requirement of agricultural green development, reflecting environmental benefits that include five secondary indicators, i.e., pesticide application intensity, fertilizer application intensity, agricultural mulch film usage, rural sanitary toilet penetration rate, and the number of natural reserves in various regions. Industrial extension and integration is new requirements for the green development of agriculture, reflecting social benefits, i.e., two secondary indicators of the number of green food per unit area and the Engel coefficient of rural residents.

## Methods

In evaluation research, scholars often use the analytic hierarchy process, fuzzy comprehensive evaluation method, DEA, principal component analysis method, entropy method, and so on. Among them, evaluation methods, such as analytic hierarchy process, and fuzzy comprehensive evaluation methods are subjective weighting evaluation methods, which do not require a high sample size, but their weights are given by experts or decision-makers based on subjective experience, which has great subjective arbitrariness. However, the DEA method, the principal component analysis method, entropy method, and network analysis have very high requirements on the sample size (29–31). If the sample size is small, it cannot be used or the evaluation results are greatly biased. The entropy law may affect the weight obtained due to the fluctuation of the data. Therefore, many scholars combine different evaluation methods to improve the defects of different evaluation methods. For example, Zhou et al. (32) combined the entropy method with neural network in the evaluation; Li et al. (33) combined the entropy method with TOPSIS; Zhong et al. (34) combined the entropy method with the analytic hierarchy process; and Ming-Lang et al. (35) used bibliometric analysis and fuzzy Delphi method.

Because of the above analysis, this paper adopted the gray relational analysis method of entropy method. The reason is that objective weighting by entropy method can remove the subjective influence; while the gray correlation analysis method can deal with incompletely clear gray systems, and the evaluation accuracy of small sample indicators is relatively high.

### The Basic Principle of the Entropy Method

Information entropy was proposed by Shannon in 1948 and used to analyze and study the uncertainty of information through probabilistic methods. The entropy method is an objective weighting method that determines the weight of indicators based on the amount of information contained in each indicator, which can avoid bias due to subjective factors (36). Evaluation index is n items, the original index data matrix X = (X_ij_)_m×n_ is obtained. For a certain index X_j_, the greater the gap between the index value X_ij_, the greater the role that the index plays in the comprehensive evaluation; if the index values of a certain index are all equal, the index does not affect the comprehensive evaluation.

The calculation steps are:

The first step is to list the matrix,


$$\begin{array}{l} A={\left(\begin{array}{ccc} X_{11} & \cdots & X_{1m}\\ \vdots & \vdots & \vdots \\ X_{n1} & \cdots & X_{nm} \end{array}\right)}_{n\times m}, \end{array}$$

X_ij_ is the value of the j-th index in the i-th scheme.

The second step is to standardize data processing. The decision matrix X = (X_ij_)_m×n_ is standardized by linear interpolation: the positive index is standardized by formula (1), and the negative index is standardized by formula (2).


(1) $$\begin{array}{l} X_{ij}^{{}^{\prime }}=\frac{X_{ij}-\min\left(X_{1j},X_{2j},\cdots \;,X_{nj}\right)}{\max\left(X_{1j},X_{2j},\cdots \;,X_{nj}\right)-\min\left(X_{1j},X_{2j},\cdots \;,X_{nj}\right)}\text{+}1 \end{array}$$

*i*=1,2……*n*;*j*=1,2……*m*


(2) $$\begin{array}{l} X_{ij}^{{}^{\prime }}=\frac{\max\left(X_{1j},X_{2j},\cdots \;,X_{nj}\right)-X_{ij}}{\max\left(X_{1j},X_{2j},\cdots \;,X_{nj}\right)-\min\left(X_{1j},X_{2j},\cdots \;,X_{nj}\right)}\text{+1} \end{array}$$

i=1,2……n;j=1,2……m

The third step is to calculate the proportion of the i-th scheme under the j-th index in the index,


(3) $$\begin{array}{l} P_{ij}=\frac{X_{ij}}{\overset{n}{\underset{i=1}{\sum }}X_{ij}} \end{array}$$

j=1,2……m

The fourth step is to calculate the information entropy value of the j-th index,


(4) $$\begin{array}{l} e_{j}=-k\times \overset{n}{\underset{i=1}{\sum }}P_{ij}\log\left(P_{ij}\right) \end{array}$$

K > 0, ln is the natural logarithm, ej ≥ 0. The constant k is related to the number of samples m, generally, let k = 1/lnn, then 0 ≤ e ≤ 1

The fifth step is to calculate the index information entropy utility of the j-th index.

For the j-th index, the greater the difference of the index value Xij, the greater the effect on program evaluation. Therefore, the smaller the entropy value ej, the larger the gj = 1–ej, the more important the index.

The sixth step is to calculate the weights,


(5) $$\begin{array}{l} W_{j}=\frac{g_{j}}{\overset{m}{\underset{j=1}{\sum }}g_{j}} \end{array}$$

j=1,2……m

The seventh step is to calculate the comprehensive score of each indicator,


(6) $$\begin{array}{l} S_{i}=\overset{m}{\underset{j=1}{\sum }}W_{j}\times P_{ij} \end{array}$$

*i*=1,2……*n*

### Gray Relational Analysis

Gray relational analysis is an important branch of gray system theory. Its basic idea is as follows: first determine the best plan according to the research problem, that is, obtain the optimal sequence of numbers. Secondly, the degree of relevance is judged by the degree of similarity between the sequence curve and geometric shape of the scheme and the curve and geometric shape of the ideal optimal sequence. The closer the curve is to the geometric shape, the greater the degree of relevance, and the closer the solution is to the ideal optimal, and vice versa. Finally, sort according to the degree of relevance to judge the pros and cons of the schemes (2).

The gray comprehensive evaluation is mainly based on the following models:


(7) $$\begin{array}{l} R=E\times W \end{array}$$

In formula (7), $R={\left[r_{1},r_{2},\cdots \;,r_{m}\right]}^{T}$ is the comprehensive evaluation result vector of m evaluated objects; $W={\left[w_{1},w_{2},\cdots w_{n}\right]}^{T}$ is the weight vector of *n* evaluation indicators, and $\sum_{j=1}^{n}w_{j}=1$; E is the evaluation matrix of each index, namely:


$$\begin{array}{l} E=\left[\begin{array}{cccc} \xi_{1}\left(1\right) & \xi_{1}\left(2\right) & \cdots & \xi_{1}\left(n\right)\\ \xi_{2}\left(1\right) & \xi_{2}\left(2\right) & \cdots & \xi_{2}\left(n\right)\\ \cdots & \cdots & \cdots & \cdots \\ \xi_{m}\left(1\right) & \xi_{m}\left(2\right) & \cdots & \xi_{m}\left(n\right) \end{array}\right] \end{array}$$

ξ_*i*_(*k*) is the correlation coefficient between the k-th index and the k-th optimal index, sorted according to the value of R.

First, determine the optimal set of indicators(F^*^), set $F^{*}=\left[j_{1}^{*},j_{2}^{*},\cdots \;,j_{n}^{*}\right]$, where $j_{k}^{*}\left(k=1,2,\cdots n,\right)$ is the optimal value of the k-th index. When selecting the optimal value, we can take the maximum value according to a certain index in the plan, if the larger the better, and vice versa, take the minimum value; or according to the best value recognized by the evaluation. In addition, when considering the optimal value, both advancement and feasibility must be considered. If the optimal value index is selected too high or too low, it is not in line with reality, and the evaluation results may be incorrect.

After selecting the final index, construct the optimal index matrix D:


$$\begin{array}{l} D\text{=}\left[\begin{array}{cccc} j_{1}^{*} & j_{2}^{*} & \cdots & j_{n}^{*}\\ j_{1}^{1} & j_{2}^{1} & \cdots & j_{n}^{1}\\ \cdots & \cdots & \cdots & \cdots \\ j_{1}^{m} & j_{2}^{m} & \cdots & j_{n}^{m} \end{array}\right] \end{array}$$

$j_{k}^{i}$ is the original data of the k-th indicator in the i-th scheme.

Second, standardize the index data. In order to eliminate the dimension and order of magnitude and ensure the reliability of the data, the indicators are standardized. The standardized data are $C_{k}^{i}\in$ (0,1), then matrix D→ C.


$$\begin{array}{l} C=\left[\begin{array}{cccc} C_{1}^{*} & C_{2}^{*} & \cdots & C_{n}^{*}\\ C_{1}^{1} & C_{2}^{1} & \cdots & C_{n}^{1}\\ \cdots & \cdots & \cdots & \cdots \\ C_{1}^{m} & C_{2}^{m} & \cdots & C_{n}^{m} \end{array}\right] \end{array}$$

Third, calculate the comprehensive evaluation results. According to the gray system theory, with $\left\{C^{*}\right\}=\left[C_{1}^{*},C_{2}^{*},\cdots C_{n}^{*}\right]$ as the reference sequence and the sequence $\left\{C^{i}\right\}=\left[C_{1}^{i},C_{2}^{i},\cdots C_{n}^{i}\right]$ as the comparison sequence, the correlation coefficient between the k-th index of the i-th scheme and the k-th optimal index is obtained through the correlation analysis method, namely:


(8) $$\begin{array}{l} \xi_{i}\left(k\right)=\frac{\underset{i}{\min}\underset{k}{\min}|C_{k}^{*}-C_{k}^{i}|+\rho \underset{i}{\max}\underset{k}{\max}|C_{k}^{*}-C_{k}^{i}|}{\left|C_{k}^{*}-C_{k}^{i}|+\rho \underset{i}{\max}\underset{k}{\max}|C_{k}^{*}-C_{k}^{i}\right|} \end{array}$$

Where ρϵ[0,1], Generally take ρ = 0.5.

Obtain the matrix E from ξ_*i*_(*k*), and then calculate the evaluation result as R = E × W,


(9) $$\begin{array}{l} r_{i}=\overset{n}{\underset{k=1}{\sum }}W\left(k\right)\times \xi_{i}\left(k\right) \end{array}$$

If the degree of relevance *r*_*i*_ is the largest, it means that {*C*^*i*^} is the closest to the optimal index {C^*^}, and the i-th scheme is better than other schemes, and the order of each scheme is arranged in turn.

## Results and Discussion

### Determine the Index Weight

According to the calculation steps of the entropy method, the weights of 15 rating indicators in the evaluation index system of agricultural green development level and the weights of each layer are calculated. The specific results are shown in Table 3.

**Table 3:** Index weights of each layer in the evaluation index system of agricultural green development level in the three northeastern provinces.

**A-level indicator**	**WA_**n**_**	**B-level indicator**	**WA_**n**_B_**i**_**	**WBi**
Agricultural development (A_1_)	0.3086	Per capita agriculture, forestry, animal husbandry and fishery output value (B_1_)	0.2071	0.0639
		Unit planting area yield of grain (B_2_)	0.1440	0.0444
		Unit planting area total agricultural output value (B_3_)	0.1965	0.0607
		Unit planting area total power of agricultural machinery (B_4_)	0.1993	0.0615
		Labor productivity (B_5_)	0.2529	0.0781
Ecological resource protection (A_2_)	0.1560	Forest coverage rate (B_6_)	0.3828	0.0597
		Area of soil erosion under control (B_7_)	0.3405	0.0531
		Water-saving irrigation efficiency (B_8_)	0.2766	0.0432
Environment-friendly (A_3_)	0.3802	Pesticide application intensity (B_9_)	0.1114	0.0423
		Fertilizer application intensity (B_10_)	0.3247	0.1234
		Agricultural film application intensity (B_11_)	0.2406	0.0915
		Popularization rate of rural sanitary toilets (B_12_)	0.0506	0.0192
		Number of nature reserves at all levels (national and provincial) (B_13_)	0.2728	0.1037
Industrial extension & integration (A_4_)	0.1552	Number of green food labels per unit area (B_14_)	0.5314	0.0825
	0.3086	Engel coefficient of rural residents (B_15_)	0.4686	0.0727

*Source, Calculated by entropy method*.

### Calculation of Correlation Coefficient and Correlation Degree

#### Calculate the Correlation Coefficient

Based on the previous research, the optimal sequence is determined. According to the formulas and steps of the gray correlation analysis, first, calculate the index correlation coefficient matrix E, and take ρ = 0.5. The calculation results are shown in Supplementary Table 1.

#### Calculating the Index Relevance

Calculate the index weights of each layer through the correlation coefficient matrix calculated in this section and the previous section. According to the calculation formula and calculation steps of the gray correlation method, calculate the correlation degree of the B-level and A-level indicators of each province (see Supplementary Table 2 and Table 4).

**Table 4:** Correlation degree of A-level indicators.

**ID**	**Year**	**RA_**1**_**	**RA_**2**_**	**RA_**3**_**	**RA_**4**_**
1	2009	0.3435	0.5699	0.8501	0.4477
1	2010	0.3592	0.5830	0.8363	0.4060
1	2011	0.3874	0.5989	0.8495	0.3892
1	2012	0.4238	0.6157	0.8423	0.3634
1	2013	0.4686	0.6283	0.8508	0.3957
1	2014	0.4983	0.6231	0.9037	0.5633
1	2015	0.5167	0.6310	0.9161	0.6006
1	2016	0.5380	0.6648	0.9189	0.6233
1	2017	0.5920	0.6800	0.9148	0.7192
1	2018	0.6196	0.7495	0.9359	0.7445
1	2019	0.6934	0.7724	0.9853	0.7172
2	2009	0.3752	0.4591	0.5026	0.3920
2	2010	0.3909	0.4598	0.5035	0.3723
2	2011	0.4321	0.4671	0.5011	0.3795
2	2012	0.4546	0.4676	0.4952	0.3579
2	2013	0.4755	0.4489	0.4999	0.4061
2	2014	0.4587	0.4400	0.4973	0.4871
2	2015	0.4730	0.4372	0.4974	0.5155
2	2016	0.4863	0.4429	0.5033	0.5410
2	2017	0.4902	0.4566	0.5061	0.5894
2	2018	0.4489	0.4908	0.5210	0.6392
2	2019	0.4789	0.4948	0.5340	0.6381
3	2009	0.4342	0.6350	0.3854	0.6227
3	2010	0.4602	0.6438	0.3791	0.5727
3	2011	0.5076	0.6796	0.3714	0.5493
3	2012	0.5633	0.7448	0.3700	0.5301
3	2013	0.6036	0.5041	0.3800	0.5112
3	2014	0.5712	0.5213	0.3851	0.6637
3	2015	0.6143	0.5253	0.3956	0.7038
3	2016	0.5277	0.5572	0.4037	0.8723
3	2017	0.5483	0.5872	0.4106	0.9215
3	2018	0.5441	0.6166	0.4235	0.9725
3	2019	0.6177	0.6293	0.4406	0.8629

*id “1,” “2,” and “3” represent Heilongjiang Province, Jilin Province, and Liaoning Province respectively*.

*Data source: calculated*.

Furthermore, the comprehensive correlation degree of the agricultural green development level of the three provinces in Northeast China from 2009 to 2019 was obtained through calculation. See Table 5 for details.

**Table 5:** Comprehensive correlation degree of agricultural green development level in the three northeastern provinces.

**Year**	**Heilongjiang province**	**Jilin province**	**Liaoning province**
2009	0.5876	0.4393	0.4762
2010	0.5827	0.4416	0.4755
2011	0.5964	0.4556	0.4891
2012	0.6035	0.4571	0.5130
2013	0.6275	0.4698	0.4888
2014	0.6820	0.4748	0.5070
2015	0.6994	0.4833	0.5312
2016	0.7158	0.4945	0.5386
2017	0.7482	0.5064	0.5599
2018	0.7795	0.5124	0.5760
2019	0.8204	0.5271	0.5902

*Data source: calculated*.

### Analysis of Evaluation Results

#### Analysis of Overall Development Level

**Figure 1:**
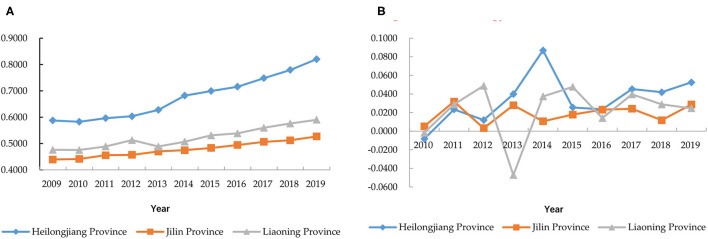
Agricultural green development levels in the three northeastern provinces (2009–2019). **(A)** Comprehensive correlation degree; **(B)** the growth rate of the comprehensive correlation degree.

From the empirical results, as shown in Figure 1A, during the period 2009–2019, the level of agricultural green development in the three provinces of Northeast China shows an overall increasing trend. The average comprehensive correlation scores were 0.6766 in Heilongjiang Province, 0.4784 in Jilin Province, and 0.5223 in Liaoning Province. According to the comprehensive correlation, the level of agricultural green development in the three northeast provinces is Heilongjiang Province > Liaoning Province > Jilin Province. Scholars have the same empirical results (17, 18, 37), but fluctuations in the level of green development in agriculture vary slightly from province to province due to different research perspectives, indicator choices, and research methods. The fluctuating rise in the level of green agricultural development in the three northeastern provinces is mainly due to the high priority given to green agricultural development by governments at all levels, for example, documents, such as “the Northeast Blackland Conservation Planning Outline (2017–2030),” have been issued to guide agricultural green development (38). Specific practices include promoting the use of organic fertilizers, with the main measure being the return of straw to the fields (39), with the rate of returning straw to the fields in the three northeastern provinces reaching about 80% by 2020, and the effective use of livestock and poultry breeding waste; promoting pilot crop rotation and the conversion of grain to feed and promoting the rotation of maize with soybeans, mixed cereals, yams, and oil crops to achieve nitrogen fixation and fertilization of fields and the combination of use and feeding (40); and promoting that the Government should also promote the reduction of fertilizers and pesticides and increase their efficiency, carry out zero growth in fertilizer and pesticide use, promote mechanized precision fertilizer application and spraying, and promote efficient new fertilizers and water-fertilizer integration technology (41).

**Figure 2:**
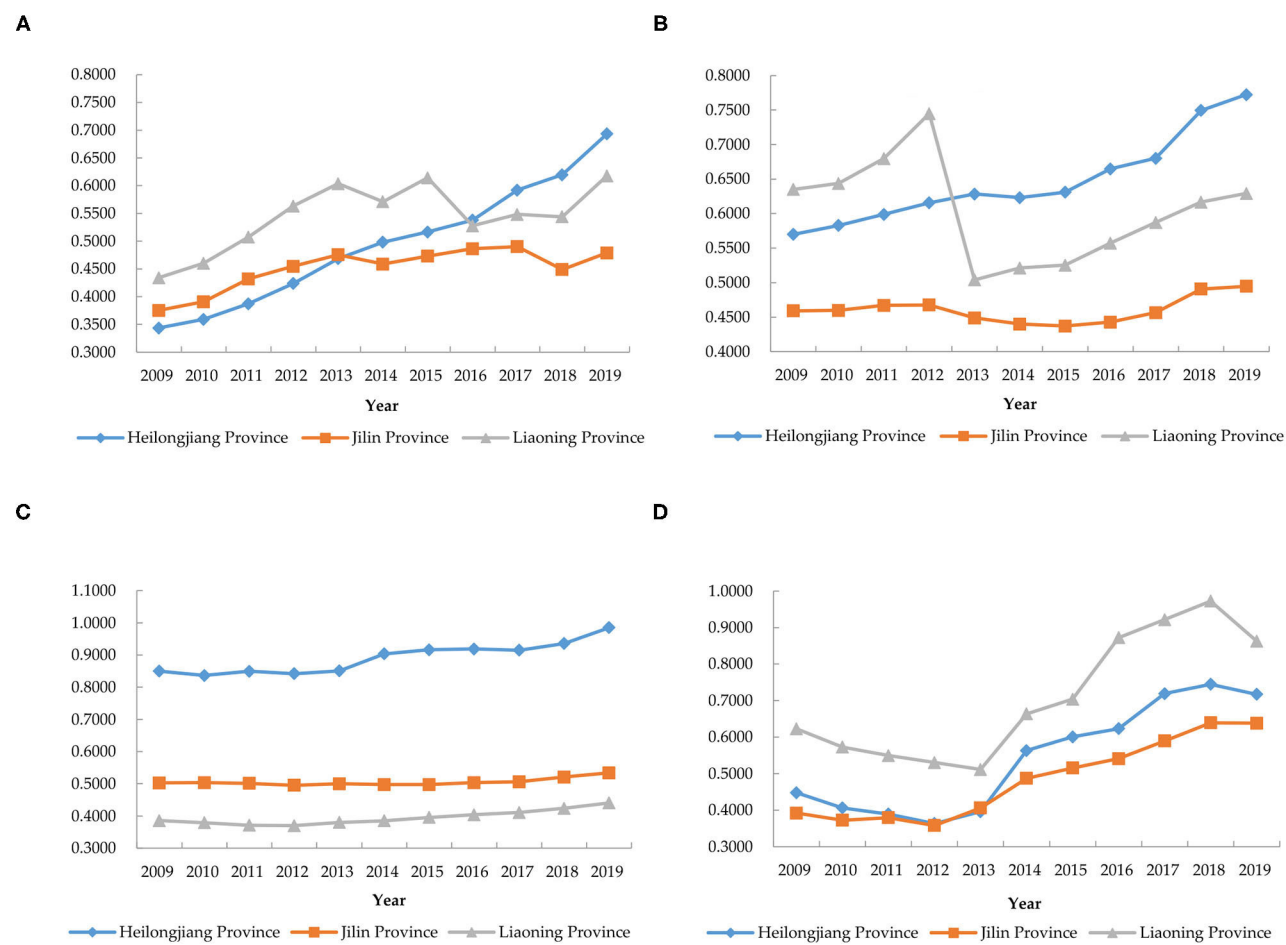
The correlation degree of each dimension of agricultural green development in the three northeastern provinces of China (2009–2019). **(A)** Agricultural development; **(B)** ecological resource protection; **(C)** environment-friendly; **(D)** industrial extension and integration.

Specifically, as shown in Figure 1B, Heilongjiang Province has the highest level of agricultural green development, with a comprehensive correlation degree growth rate ranging from −0.0082 to 0.0868, with an average growth rate of 0.0342. The level of green development in agriculture in Heilongjiang province has generally maintained its growth, and Chinese scholar Cui (42) reached similar conclusions. In 2014, the level of green agricultural development in Heilongjiang Province was increased the most, and further analysis revealed that the dimension of industrial extension and integration was increased more in that year, mainly due to the larger increase in the number of green food labels per unit area, from 0.0992/10^3^ha in 2013 to 0.1038/10^3^ha in 2013. An analysis of Heilongjiang's policies reveals that the Heilongjiang Province “Two Plain” Modern Agriculture Comprehensive Reform Experiment Overall Implementation Plan was approved in 2013, and Heilongjiang Province launched a modern agriculture comprehensive reform experiment first, with the cities and counties involved in the document actively implementing the plan to promote rapid agricultural development (43). Liaoning Province ranked second in terms of the level of green development of agriculture, with a range of growth rates of −0.0472 to 0.0487 in terms of integrated relevance and an average growth rate of 0.0221. The level of green development in agriculture in Liaoning Province was declined significantly in 2013, and such empirical results have been derived in the existing literature, such as Cui et al. (37). Further analysis revealed that Liaoning Province experiences a significant decline in the ecological resource protection dimension in 2013 as shown in Figure 2B, and the main indicators of this dimension, area of soil erosion under control (B7), and water conservation and irrigation efficiency (B8) have decreased to a greater extent. The main reason is that Liaoning Province began to implement precise erosion control after 2012, and the area under control declined (44). At the same time, agricultural water conservancy facilities are old, resulting in their unstable effective irrigation level. Jilin Province has the lowest level of agricultural development among the three provinces, with the growth rate of the integrated correlation ranging from 0.0052 to 0.0318, with an average growth rate of 0.0184. The level of green agricultural development in Jilin province has a fluctuating upward trend in all dimensions and overall level, but the scores of ecological resource protection dimension and industrial integration and development dimension have a larger gap compared to the other two provinces.

#### Analysis of the Development Level of Each Dimension

##### Dimensions of Agricultural Development

The weight of the agricultural development dimension in the entire evaluation index system is 0.3086. During the period from 2009 to 2019, the three provinces in Northeast China showed different trends in this dimension (see Figure 2), and the average values were 0.4946 in Heilongjiang Province, 0.4513 in Jilin Province, and 0.5447 in Liaoning Province. Specifically, within the research interval, Heilongjiang Province's correlation in the agricultural development dimension has been growing steadily, with an average growth rate of 0.0732. There is still a certain gap between 2009 and 2013 and the other two provinces. In 2014 and 2016, it surpassed Jilin Province and Liaoning Province to rank the highest among the three provinces. The relevance of the agricultural development dimension of Jilin Province is generally stable and fluctuating. Except for a decline in 2014 and 2018, other years have maintained a growth trend, with an average growth rate of 0.0260. Specific analysis revealed that the decline in unit planting area yielded to the grain of Jilin Province in 2014 led to a lower score of this dimension, mainly due to natural disasters, such as low temperature and cold damage in spring and local drought in summer and autumn in that year (45). In 2018, there was again a decline in scores due to a decline in unit planting area yield of grain, which was attributed to the supply-side influence on Jilin Province to adjust its planting structure.

Liaoning Province's agricultural development dimension correlation degree is similar to that of Jilin Province, and the average growth rate was 0.0392. Generally speaking, it shows a trend of upward volatility, but its volatility is relatively large. Liaoning Province led the three provinces in agricultural development relevance scores from 2009 to 2015. In 2014, there was a significant drop mainly due to a very severe drought, which reduced grain production by 20.1%. The score remained on an upward trend after a significant drop in 2016 due to the decline in total output values of agriculture, forestry, animal husbandry, and fishery.

##### Dimension of Ecological Resource Protection

The weight of the ecological resource protection dimension in the evaluation index system is 0.1560. During the period from 2009 to 2019, the three provinces in the northeast showed different correlations in this dimension (see Figure 2), with the average values being 0.6470 in Heilongjiang Province, 0.4604 in Jilin Province, and 0.6040 in Liaoning Province. Specifically, in the research interval, the correlation degree of the production protection dimension in Heilongjiang Province is relatively stable, and it is in a fluctuating upward trend. In 2013, the correlation degree score surpassed Liaoning Province to become the highest among the three provinces, and the correlation degree has been maintained at an average growth rate of 0.0312 in 2019. The correlation degree of the ecological resource protection dimension of Jilin Province is similar to the positive “u” type, and the lowest point was in 2015. The overall score is lower than that of Heilongjiang Province and Liaoning Province, and the average growth rate of correlation degree is 0.0079. The correlation degree of the dimension of ecological resource protection in Liaoning Province maintained a continuous growth trend after a significant decline in 2013, with an average degree of correlation of 0.0069. Through the analysis, it is found that area of soil erosion under control in Liaoning Province has dropped significantly in 2013, which is an important reason for the decline in the correlation score after that year.

##### Environment-Friendly Dimensions

The weight of the environment-friendly dimension in the evaluation index system is 0.3802. As shown in Figure 2, within the study interval, the correlation of the three northeastern provinces in this dimension shows a fluctuating and rising trend, with the average values being 0.8912 in Heilongjiang Province, 0.5056 in Jilin Province, and 0.3945 in Liaoning Province. Heilongjiang Province has a higher score in the environment-friendly dimension, followed by Jilin and Liaoning Provinces third. Through analysis, it is found that Heilongjiang Province has far more nature reserves than the other two provinces. At the same time, pesticide application intensity, fertilizer application intensity, and agricultural film application intensity are more lower than the other two provinces. Therefore, Heilongjiang Province has a much higher relevance score in the environmentally friendly dimension than the other two provinces.

##### Dimensions of Industry Extension and Integration

The weight of the industrial extension and integration dimension in the evaluation index system is 0.1552. As shown in Figure 2, within the research interval, the correlation degree between the three northeast provinces in the dimensions of industrial extension and integration shows a “decreasing-increasing-decreasing” trend. The overall trend is an increasing trend, and the average degree of correlation is 0.5427 in Heilongjiang Province, 0.4835 in Jilin Province, and 0.7075 in Liaoning Province. Liaoning's industrial extension and integration dimension correlation score has always been ahead of the other two provinces, mainly due to a large number of green foods per unit area in Liaoning Province. Heilongjiang Province's industrial extension and integration dimension correlation scores were higher than those of Jilin Province, except for 2013.

## Conclusions and Policy Implications

In the context of China's Rural Revitalization Strategy, this paper constructs an evaluation index system for the level of agricultural green development from four dimensions: agricultural development, ecological resource protection, environmental friendliness, and industrial extension and integration. Subsequently, using the data from 2009 to 2019, the entropy-gray correlation method was used to evaluate the level of agricultural green development in the three provinces of Northeast China, and the evaluation results were analyzed, and the following conclusions were drawn:

(1) The level of green development of agriculture in the three northeastern provinces has steadily improved, and good development has been achieved in agricultural development, ecological resource protection, environmental improvement, and industrial integration. However, there is still a lot of room for improvement in the scores of agricultural green development level and the correlation scores of various dimensions.
(2) There are differences in the green development of agriculture in the three northeast provinces. The level of agricultural green development in the three northeastern provinces is as follows: Heilongjiang Province > Liaoning Province > Jilin Province. The level of agricultural green development in Heilongjiang Province is significantly higher than that of the other two provinces, and it maintains a relatively stable growth trend in the four dimensions of agricultural development, ecological resource protection, environment-friendly, and industrial extension and integration. The level of agricultural green development in Liaoning Province ranks second among the three provinces, and there are certain fluctuations in all dimensions. However, Liaoning Province is significantly better than the other two provinces in terms of industrial extension and integration. The level of agricultural green development in Jilin Province is slightly weaker than the other two provinces, but both the level of agricultural green development and the development of all dimensions are relatively stable.

Based on the above research results, the following policy implications are drawn:

The first is to establish an evaluation system for agricultural green development at the national level. The implementation of dynamic monitoring and management of agricultural green development can promptly grasp the situation of agricultural green development, issue corresponding policies and provide data support and data accumulation for scientific research.

The second is to strengthen the protection of agricultural ecological resources. Increase the efficiency of agricultural ecological protection through measures, such as increasing the application of organic fertilizers, reducing the number of pesticides and increasing efficiency, and establishing a recycling mechanism for agricultural mulch films.

The third is to increase rural environmental governance. Formulate more complete and reasonable pollution management policies, strengthen environmental control, and raise industry access thresholds to continue to promote the green and clean development of agricultural production (46).

The fourth is to promote the integrated development of rural tertiary industries. Grasping the strategy of rural revitalization and speeding up the development of the integration of the three industries in rural areas. While promoting the optimization of the industrial structure, more attention should be paid to the policy preference for agriculture, to achieve green efficiency in agricultural production and stable growth of farmers' income (47).

The fifth is to vigorously support agricultural technological innovation. To be problem-oriented, vigorously carry out agricultural scientific and technological innovation, strengthen scientific and technological research on black soil conservation and other issues, and breakthrough the bottleneck of agricultural green production. At the same time, speed up the transformation and promotion of green technology achievements, strengthen the application of fine varieties and methods, and coordinate production ecology. In addition, it is necessary to strengthen the training of agricultural green production knowledge and technology to improve the scientific and technological quality of agricultural employees and the ability to apply green technology.

The sixth is to strengthen regional cooperation. Strengthen the exchange, learning, and cooperation of agricultural green production technology and production experience between regions. The provinces have made precise efforts to address the shortcomings in the green development of agriculture and promote coordinated development among regions.

## Data Availability Statement

The original contributions presented in the study are included in the article/Supplementary Material, further inquiries can be directed to the corresponding authors.

## Author Contributions

DH and XW: conceptualization, writing—original draft preparation, and funding acquisition. DH: methodology, software, investigation, and data curation. XW: writing—review and editing. Both authors have read and agreed to the published version of the manuscript.

## Funding

This research was funded by Fujian Province Young and Middle-aged Teacher Education Research Project (Social Science) (Grant No. JAS19169), Minnan Normal University President's Fund Project (Grant No. SK18017), the Fujian Provincial Social Science Planning Project (Grant No. FJ2021BF038), the Educational Research Project for Young and Middle-aged Teachers in Fujian Province (Grant No. JAT190735), the Longyan University Startup Foundation for PhD (Grant No. LB2020001), Fujian Natural Science Foundation (Grant No. 2020J05204), and the Technological Innovation Fund Project of Scientific and Technological Small and Medium-sized Enterprises in Fujian Province (Grant No. 2021C0054).

## Conflict of Interest

XW was employed by Fujian Jonorm Bio-Pharmaceutical Co., Ltd. The remaining author declares that the research was conducted in the absence of any commercial or financial relationships that could be construed as a potential conflict of interest.

## Publisher's Note

All claims expressed in this article are solely those of the authors and do not necessarily represent those of their affiliated organizations, or those of the publisher, the editors and the reviewers. Any product that may be evaluated in this article, or claim that may be made by its manufacturer, is not guaranteed or endorsed by the publisher.
