# Supplementary material for: Measurement of Agricultural Green Development Level in the Three Provinces of Northeast China Under the Background of Rural Vitalization Strategy
Source: Front Public Health. 2022 Mar 11;10:824202. doi: 10.3389/fpubh.2022.824202 (PMC8962617; doi:10.3389/fpubh.2022.824202)
Supplement: Supplementary file 1 [file Data_Sheet_1.docx]

**Supplementary Materials:** Table S1: Correlation coefficient matrix, TableS2: Correlation degree of B-level indicators .

**Table S1.** Correlation coefficient matrix.

| **ID** | **year** | **B_1_** | **B_2_** | **B_3_** | **B_4_** | **B_5_** | **B_6_** | **B_7_** | **B_8_** | **B_9_** | **B_10_** | **B_11_** | **B_12_** | **B_13_** | **B_14_** | **B_15_** |
| --- | --- | --- | --- | --- | --- | --- | --- | --- | --- | --- | --- | --- | --- | --- | --- | --- |
| 1 | 2009 | 0.3466 | 0.3333 | 0.3333 | 0.3333 | 0.3627 | 0.7568 | 0.5519 | 0.3333 | 0.9017 | 0.9452 | 0.9828 | 0.4905 | 0.6656 | 0.3517 | 0.5565 |
| 1 | 2010 | 0.3610 | 0.3738 | 0.3395 | 0.3415 | 0.3786 | 0.7568 | 0.5635 | 0.3664 | 0.8680 | 0.8949 | 0.9738 | 0.5421 | 0.6869 | 0.3581 | 0.4604 |
| 1 | 2011 | 0.4039 | 0.4005 | 0.3568 | 0.3498 | 0.4196 | 0.7568 | 0.5824 | 0.4007 | 0.8579 | 0.8663 | 0.9556 | 0.5843 | 0.7818 | 0.3612 | 0.4211 |
| 1 | 2012 | 0.4626 | 0.4148 | 0.3798 | 0.3613 | 0.4807 | 0.7568 | 0.6016 | 0.4380 | 0.8540 | 0.8466 | 0.9322 | 0.5927 | 0.7993 | 0.3702 | 0.3556 |
| 1 | 2013 | 0.5416 | 0.4355 | 0.4074 | 0.3675 | 0.5550 | 0.8746 | 0.4554 | 0.5004 | 0.8498 | 0.8538 | 0.9400 | 0.6225 | 0.8113 | 0.3757 | 0.4183 |
| 1 | 2014 | 0.5919 | 0.4469 | 0.4156 | 0.3735 | 0.6137 | 0.8746 | 0.4635 | 0.4716 | 0.8467 | 0.8542 | 0.9514 | 0.6503 | 0.9908 | 0.3801 | 0.7711 |
| 1 | 2015 | 0.6378 | 0.4504 | 0.4099 | 0.3797 | 0.6461 | 0.8746 | 0.4743 | 0.4868 | 0.8786 | 0.8624 | 0.9609 | 0.6769 | 1.0000 | 0.3877 | 0.8421 |
| 1 | 2016 | 0.6830 | 0.4382 | 0.4125 | 0.3881 | 0.6917 | 0.8746 | 0.5100 | 0.5651 | 0.8760 | 0.8663 | 0.9593 | 0.7719 | 0.9908 | 0.4494 | 0.8205 |
| 1 | 2017 | 0.7826 | 0.4396 | 0.4286 | 0.3959 | 0.8040 | 0.8746 | 0.5382 | 0.5853 | 0.8744 | 0.8691 | 0.9720 | 0.6163 | 0.9908 | 0.4983 | 0.9697 |
| 1 | 2018 | 0.8281 | 0.4447 | 0.4373 | 0.4060 | 0.8586 | 1.0000 | 0.5900 | 0.5992 | 0.9231 | 0.8943 | 0.9785 | 0.7322 | 0.9908 | 0.5192 | 1.0000 |
| 1 | 2019 | 1.0000 | 0.4394 | 0.4437 | 0.4156 | 1.0000 | 1.0000 | 0.6562 | 0.6002 | 1.0000 | 0.9997 | 1.0000 | 0.7610 | 0.9908 | 0.5317 | 0.9275 |
| 2 | 2009 | 0.3333 | 0.4627 | 0.3687 | 0.4126 | 0.3351 | 0.4714 | 0.4503 | 0.4530 | 0.6689 | 0.3898 | 0.7528 | 0.5829 | 0.3333 | 0.3664 | 0.4211 |
| 2 | 2010 | 0.3375 | 0.5372 | 0.3748 | 0.4253 | 0.3369 | 0.4714 | 0.4535 | 0.4516 | 0.6733 | 0.3806 | 0.7547 | 0.6304 | 0.3354 | 0.3647 | 0.3810 |
| 2 | 2011 | 0.3616 | 0.7131 | 0.3888 | 0.4500 | 0.3493 | 0.4729 | 0.4578 | 0.4705 | 0.6565 | 0.3881 | 0.7363 | 0.6589 | 0.3354 | 0.3477 | 0.4156 |
| 2 | 2012 | 0.3696 | 0.8013 | 0.4005 | 0.4718 | 0.3554 | 0.4714 | 0.4621 | 0.4693 | 0.6186 | 0.3725 | 0.7471 | 0.6696 | 0.3365 | 0.3375 | 0.3810 |
| 2 | 2013 | 0.3774 | 0.9171 | 0.4016 | 0.4793 | 0.3588 | 0.5599 | 0.3333 | 0.4375 | 0.6404 | 0.3690 | 0.7528 | 0.6807 | 0.3418 | 0.3333 | 0.4885 |
| 2 | 2014 | 0.3788 | 0.7899 | 0.3995 | 0.4861 | 0.3598 | 0.5599 | 0.3388 | 0.3986 | 0.5988 | 0.3544 | 0.7740 | 0.6901 | 0.3462 | 0.3348 | 0.6598 |
| 2 | 2015 | 0.3840 | 0.8503 | 0.3959 | 0.5137 | 0.3591 | 0.5599 | 0.3444 | 0.3816 | 0.5895 | 0.3554 | 0.7740 | 0.6882 | 0.3496 | 0.3499 | 0.7033 |
| 2 | 2016 | 0.3786 | 0.9978 | 0.3761 | 0.5043 | 0.3547 | 0.5599 | 0.3536 | 0.3908 | 0.6153 | 0.3522 | 0.7722 | 0.7768 | 0.3496 | 0.3693 | 0.7356 |
| 2 | 2017 | 0.3738 | 1.0000 | 0.3702 | 0.5374 | 0.3515 | 0.5599 | 0.3632 | 0.4285 | 0.6315 | 0.3563 | 0.7658 | 0.7993 | 0.3496 | 0.4293 | 0.7711 |
| 2 | 2018 | 0.3819 | 0.6379 | 0.3799 | 0.5651 | 0.3583 | 0.6538 | 0.3741 | 0.4089 | 0.6775 | 0.3644 | 0.7946 | 0.8042 | 0.3496 | 0.4884 | 0.8101 |
| 2 | 2019 | 0.4026 | 0.7397 | 0.3814 | 0.6010 | 0.3727 | 0.6538 | 0.3858 | 0.4090 | 0.7011 | 0.3695 | 0.8156 | 0.8333 | 0.3577 | 0.5126 | 0.7805 |
| 3 | 2009 | 0.3349 | 0.4279 | 0.4559 | 0.6485 | 0.3333 | 0.3333 | 0.8548 | 0.7820 | 0.4339 | 0.3496 | 0.3623 | 0.4718 | 0.4127 | 0.8358 | 0.3810 |
| 3 | 2010 | 0.3461 | 0.4785 | 0.5105 | 0.6685 | 0.3399 | 0.3333 | 0.8816 | 0.7807 | 0.3671 | 0.3434 | 0.3667 | 0.5118 | 0.4127 | 0.7693 | 0.3497 |
| 3 | 2011 | 0.3623 | 0.6261 | 0.5602 | 0.7211 | 0.3500 | 0.3333 | 0.9305 | 0.8501 | 0.4317 | 0.3462 | 0.3333 | 0.3333 | 0.4175 | 0.7397 | 0.3333 |
| 3 | 2012 | 0.3764 | 0.6349 | 0.6344 | 0.8946 | 0.3593 | 0.3333 | 1.0000 | 0.9999 | 0.3333 | 0.3391 | 0.3372 | 0.5129 | 0.4241 | 0.6925 | 0.3459 |
| 3 | 2013 | 0.3855 | 0.7481 | 0.6768 | 0.9570 | 0.3648 | 0.4384 | 0.5432 | 0.5468 | 0.4262 | 0.3333 | 0.3394 | 0.5433 | 0.4224 | 0.5279 | 0.4923 |
| 3 | 2014 | 0.3883 | 0.4563 | 0.6768 | 1.0000 | 0.3664 | 0.4384 | 0.5688 | 0.5776 | 0.4312 | 0.3406 | 0.3442 | 0.5617 | 0.4224 | 0.5771 | 0.7619 |
| 3 | 2015 | 0.3944 | 0.5537 | 0.8204 | 0.9930 | 0.3702 | 0.4384 | 0.5850 | 0.5722 | 0.4453 | 0.3519 | 0.3533 | 0.6240 | 0.4224 | 0.6444 | 0.7711 |
| 3 | 2016 | 0.3812 | 0.6618 | 0.7035 | 0.6205 | 0.3615 | 0.4384 | 0.6080 | 0.6589 | 0.4579 | 0.3524 | 0.3632 | 0.6960 | 0.4241 | 0.8353 | 0.9143 |
| 3 | 2017 | 0.3862 | 0.6966 | 0.7378 | 0.6560 | 0.3644 | 0.4384 | 0.6373 | 0.7316 | 0.4460  0.4623 | 0.3539 | 0.3853 | 0.7441 | 0.4241 | 0.9042 | 0.9412 |
| 3 | 2018 | 0.3963 | 0.5961 | 0.8318 | 0.5964 | 0.3707 | 0.4879 | 0.6665 | 0.7332 |  | 0.3567 | 0.4023 | 0.8643 | 0.4241 | 1.0001 | 0.9412 |
| 3 | 2019 | 0.4145 | 0.7705 | 1.0000 | 0.6394 | 0.3828 | 0.4879 | 0.6952 | 0.7438 | 0.4893 | 0.3707 | 0.4137 | 1.0000 | 0.4241 | 0.7814 | 0.9552 |

Note: id “1”, “2”, and “3” represent Heilongjiang Province, Jilin Province and Liaoning Province respectively; data source: calculated based on grey relational analysis.

**Table S2.** Correlation degree of B-level indicators.

| **ID** | **B_1_** | **B_2_** | **B_3_** | **B_4_** | **B_5_** | **B_6_** | **B_7_** | **B_8_** | **B_9_** | **B_10_** | **B_11_** | **B_12_** | **B_13_** | **B_14_** | **B_15_** |
| --- | --- | --- | --- | --- | --- | --- | --- | --- | --- | --- | --- | --- | --- | --- | --- |
| 1 | 0.0222 | 0.0148 | 0.0202 | 0.0205 | 0.0283 | 0.0452 | 0.0293 | 0.0144 | 0.0382 | 0.1166 | 0.0899 | 0.0094 | 0.0690 | 0.0290 | 0.0405 |
| 1 | 0.0231 | 0.0166 | 0.0206 | 0.0210 | 0.0296 | 0.0452 | 0.0299 | 0.0158 | 0.0367 | 0.1104 | 0.0891 | 0.0104 | 0.0712 | 0.0295 | 0.0335 |
| 1 | 0.0258 | 0.0178 | 0.0216 | 0.0215 | 0.0328 | 0.0452 | 0.0309 | 0.0173 | 0.0363 | 0.1069 | 0.0874 | 0.0112 | 0.0811 | 0.0298 | 0.0306 |
| 1 | 0.0296 | 0.0184 | 0.0230 | 0.0222 | 0.0375 | 0.0452 | 0.0320 | 0.0189 | 0.0362 | 0.1045 | 0.0853 | 0.0114 | 0.0829 | 0.0305 | 0.0259 |
| 1 | 0.0346 | 0.0194 | 0.0247 | 0.0226 | 0.0433 | 0.0522 | 0.0242 | 0.0216 | 0.0360 | 0.1054 | 0.0860 | 0.0120 | 0.0841 | 0.0310 | 0.0304 |
| 1 | 0.0378 | 0.0199 | 0.0252 | 0.0230 | 0.0479 | 0.0522 | 0.0246 | 0.0204 | 0.0358 | 0.1054 | 0.0870 | 0.0125 | 0.1028 | 0.0313 | 0.0561 |
| 1 | 0.0408 | 0.0200 | 0.0249 | 0.0234 | 0.0504 | 0.0522 | 0.0252 | 0.0210 | 0.0372 | 0.1064 | 0.0879 | 0.0130 | 0.1037 | 0.0320 | 0.0612 |
| 1 | 0.0437 | 0.0195 | 0.0250 | 0.0239 | 0.0540 | 0.0522 | 0.0271 | 0.0244 | 0.0371 | 0.1069 | 0.0877 | 0.0148 | 0.1028 | 0.0371 | 0.0597 |
| 1 | 0.0500 | 0.0195 | 0.0260 | 0.0244 | 0.0628 | 0.0522 | 0.0286 | 0.0253 | 0.0370 | 0.1073 | 0.0889 | 0.0119 | 0.1028 | 0.0411 | 0.0705 |
| 1 | 0.0529 | 0.0198 | 0.0265 | 0.0250 | 0.0670 | 0.0597 | 0.0314 | 0.0259 | 0.0391 | 0.1104 | 0.0895 | 0.0141 | 0.1028 | 0.0428 | 0.0727 |
| 1 | 0.0639 | 0.0195 | 0.0269 | 0.0256 | 0.0781 | 0.0597 | 0.0349 | 0.0259 | 0.0423 | 0.1234 | 0.0915 | 0.0146 | 0.1028 | 0.0438 | 0.0675 |
| 2 | 0.0213 | 0.0206 | 0.0224 | 0.0254 | 0.0262 | 0.0282 | 0.0239 | 0.0196 | 0.0283 | 0.0481 | 0.0688 | 0.0112 | 0.0346 | 0.0302 | 0.0306 |
| 2 | 0.0216 | 0.0239 | 0.0227 | 0.0262 | 0.0263 | 0.0282 | 0.0241 | 0.0195 | 0.0285 | 0.0470 | 0.0690 | 0.0121 | 0.0348 | 0.0301 | 0.0277 |
| 2 | 0.0231 | 0.0317 | 0.0236 | 0.0277 | 0.0273 | 0.0283 | 0.0243 | 0.0203 | 0.0278 | 0.0479 | 0.0673 | 0.0127 | 0.0348 | 0.0287 | 0.0302 |
| 2 | 0.0236 | 0.0356 | 0.0243 | 0.0290 | 0.0277 | 0.0282 | 0.0246 | 0.0203 | 0.0262 | 0.0460 | 0.0683 | 0.0129 | 0.0349 | 0.0278 | 0.0277 |
| 2 | 0.0241 | 0.0408 | 0.0244 | 0.0295 | 0.0280 | 0.0334 | 0.0177 | 0.0189 | 0.0271 | 0.0455 | 0.0689 | 0.0131 | 0.0355 | 0.0275 | 0.0355 |
| 2 | 0.0242 | 0.0351 | 0.0242 | 0.0299 | 0.0281 | 0.0334 | 0.0180 | 0.0172 | 0.0253 | 0.0437 | 0.0708 | 0.0133 | 0.0359 | 0.0276 | 0.0480 |
| 2 | 0.0245 | 0.0378 | 0.0240 | 0.0316 | 0.0280 | 0.0334 | 0.0183 | 0.0165 | 0.0250 | 0.0439 | 0.0708 | 0.0132 | 0.0363 | 0.0289 | 0.0511 |
| 2 | 0.0242 | 0.0443 | 0.0228 | 0.0310 | 0.0277 | 0.0334 | 0.0188 | 0.0169 | 0.0260 | 0.0435 | 0.0706 | 0.0149 | 0.0363 | 0.0305 | 0.0535 |
| 2 | 0.0239 | 0.0444 | 0.0225 | 0.0331 | 0.0274 | 0.0334 | 0.0193 | 0.0185 | 0.0267 | 0.0440 | 0.0700 | 0.0154 | 0.0363 | 0.0354 | 0.0561 |
| 2 | 0.0244 | 0.0284 | 0.0230 | 0.0348 | 0.0280 | 0.0391 | 0.0199 | 0.0176 | 0.0287 | 0.0450 | 0.0727 | 0.0155 | 0.0363 | 0.0403 | 0.0589 |
| 2 | 0.0257 | 0.0329 | 0.0231 | 0.0370 | 0.0291 | 0.0391 | 0.0205 | 0.0177 | 0.0297 | 0.0456 | 0.0746 | 0.0160 | 0.0371 | 0.0423 | 0.0568 |
| 3 | 0.0214 | 0.0190 | 0.0277 | 0.0399 | 0.0260 | 0.0199 | 0.0454 | 0.0338 | 0.0184 | 0.0431 | 0.0331 | 0.0091 | 0.0428 | 0.0689 | 0.0277 |
| 3 | 0.0221 | 0.0213 | 0.0310 | 0.0411 | 0.0265 | 0.0199 | 0.0468 | 0.0337 | 0.0155 | 0.0424 | 0.0335 | 0.0098 | 0.0428 | 0.0634 | 0.0254 |
| 3 | 0.0232 | 0.0278 | 0.0340 | 0.0444 | 0.0273 | 0.0199 | 0.0494 | 0.0367 | 0.0183 | 0.0427 | 0.0305 | 0.0064 | 0.0433 | 0.0610 | 0.0242 |
| 3 | 0.0241 | 0.0282 | 0.0385 | 0.0550 | 0.0280 | 0.0199 | 0.0531 | 0.0432 | 0.0141 | 0.0419 | 0.0308 | 0.0099 | 0.0440 | 0.0571 | 0.0252 |
| 3 | 0.0246 | 0.0332 | 0.0410 | 0.0589 | 0.0285 | 0.0262 | 0.0289 | 0.0236 | 0.0180 | 0.0411 | 0.0310 | 0.0104 | 0.0438 | 0.0435 | 0.0358 |
| 3 | 0.0248 | 0.0203 | 0.0411 | 0.0615 | 0.0286 | 0.0262 | 0.0302 | 0.0249 | 0.0183 | 0.0420 | 0.0315 | 0.0108 | 0.0438 | 0.0476 | 0.0554 |
| 3 | 0.0252 | 0.0246 | 0.0498 | 0.0611 | 0.0289 | 0.0262 | 0.0311 | 0.0247 | 0.0189 | 0.0434 | 0.0323 | 0.0120 | 0.0438 | 0.0531 | 0.0561 |
| 3 | 0.0244 | 0.0294 | 0.0427 | 0.0382 | 0.0282 | 0.0262 | 0.0323 | 0.0284 | 0.0194 | 0.0435 | 0.0332 | 0.0134 | 0.0440 | 0.0689 | 0.0665 |
| 3 | 0.0247 | 0.0310 | 0.0448 | 0.0404 | 0.0285 | 0.0262 | 0.0339 | 0.0316 | 0.0189 | 0.0437 | 0.0352 | 0.0143 | 0.0440 | 0.0746 | 0.0684 |
| 3 | 0.0253 | 0.0265 | 0.0505 | 0.0367 | 0.0289 | 0.0291 | 0.0354 | 0.0316 | 0.0196 | 0.0440 | 0.0368 | 0.0166 | 0.0440 | 0.0825 | 0.0684 |
| 3 | 0.0265 | 0.0342 | 0.0607 | 0.0393 | 0.0299 | 0.0291 | 0.0369 | 0.0321 | 0.0207 | 0.0458 | 0.0378 | 0.0192 | 0.0440 | 0.0644 | 0.0695 |

Data source: calculated
